# Supplementary material for: Mechanisms governing the pioneering and redistribution capabilities of the non-classical pioneer PU.1
Source: Nat Commun. 2020 Jan 21;11:402. doi: 10.1038/s41467-019-13960-2 (PMC6972792; doi:10.1038/s41467-019-13960-2)
Supplement: Supplementary file 7 — Source data [file 41467_2019_13960_MOESM7_ESM.zip › Source_Data/FigureS3/FigureS3J_MotifScanOutput/homerResults/motif27.similar.html]

motif27

## Information for motif27

C
A
T
G
A
G
T
C
A
C
T
G
A
T
C
G
A
C
G
T
A
G
T
C
C
G
A
T
A
T
C
G
  
Reverse Opposite:  

A
T
G
C
C
G
T
A
C
T
A
G
T
C
G
A
A
T
G
C
T
G
A
C
A
C
T
G
G
T
A
C
  

|  |  |
| --- | --- |
| p-value: | 1e-43 |
| log p-value: | -1.010e+02 |
| Information Content per bp: | 1.678 |
| Number of Target Sequences with motif | 5901.0 |
| Percentage of Target Sequences with motif | 44.71% |
| Number of Background Sequences with motif | 13780.3 |
| Percentage of Background Sequences with motif | 37.75% |
| Average Position of motif in Targets | 460.1 +/- 438.5bp |
| Average Position of motif in Background | 320.8 +/- 206.7bp |
| Strand Bias (log2 ratio + to - strand density) | -0.0 |
| Multiplicity (# of sites on avg that occur together) | 1.61 |
| Motif File: | file (matrix) reverse opposite |

### Similar de novo motifs found

|  |  |  |  |  |  |  |  |
| --- | --- | --- | --- | --- | --- | --- | --- |
| Rank | Match Score | Redundant Motif | P-value | log P-value | % of Targets | % of Background | Motif file |
| 1 | 0.801 | T A C G T A G C G C T A C T A G T C A G A T G C C G T A A T C G T A G C G T A C | 1e-22 | -50.752250 | 14.84% | 11.52% | motif file (matrix) |
| 2 | 0.773 | A G T C A C G T A C T G C T A G A G T C A G C T C T A G | 1e-16 | -37.964317 | 24.21% | 20.68% | motif file (matrix) |
